# Dopamine control of pyramidal neuron activity in the primary motor cortex via D2 receptors

**DOI:** 10.3389/fncir.2014.00013

**Published:** 2014-02-28

**Authors:** Clément Vitrac, Sophie Péron, Isabelle Frappé, Pierre-Olivier Fernagut, Mohamed Jaber, Afsaneh Gaillard, Marianne Benoit-Marand

**Affiliations:** ^1^Laboratoire de Neurosciences Expérimentales et Cliniques, INSERM, U1084Poitiers, France; ^2^Laboratoire de Neurosciences Expérimentales et Cliniques, Université de PoitiersPoitiers, France; ^3^CHU de PoitiersPoitiers, France; ^4^Institut des Maladies Neurodégénératives, UMR 5293, Université de BordeauxBordeaux, France; ^5^CNRS, Institut des Maladies Neurodégénératives, UMR 5293Bordeaux, France

**Keywords:** motor cortex, dopamine, mice, unbiased stereology, *in vivo* electrophysiology

## Abstract

The primary motor cortex (M1) is involved in fine voluntary movements control. Previous studies have shown the existence of a dopamine (DA) innervation in M1 of rats and monkeys that could directly modulate M1 neuronal activity. However, none of these studies have described the precise distribution of DA terminals within M1 functional region nor have quantified the density of this innervation. Moreover, the precise role of DA on pyramidal neuron activity still remains unclear due to conflicting results from previous studies regarding D_2_ effects on M1 pyramidal neurons. In this study we assessed in mice the neuroanatomical characteristics of DA innervation in M1 using unbiased stereological quantification of DA transporter-immunostained fibers. We demonstrated for the first time in mice that DA innervates the deep layers of M1 targeting preferentially the forelimb representation area of M1. To address the functional role of the DA innervation on M1 neuronal activity, we performed electrophysiological recordings of single neurons activity *in vivo* and pharmacologically modulated D_2_ receptor activity. Local D_2_ receptor activation by quinpirole enhanced pyramidal neuron spike firing rate without changes in spike firing pattern. Altogether, these results indicate that DA innervation in M1 can increase neuronal activity through D_2_ receptor activation and suggest a potential contribution to the modulation of fine forelimb movement. Given the demonstrated role for DA in fine motor skill learning in M1, our results suggest that altered D_2_ modulation of M1 activity may be involved in the pathophysiology of movement disorders associated with disturbed DA homeostasis.

## INTRODUCTION

The primary motor cortex (M1) is involved in fine voluntary movements control and in novel motor skill learning (Hosp et al., 2011). It integrates inputs from the premotor cortex and drives excitatory outputs to the spinal cord and the basal ganglia via glutamatergic pyramidal neurons. Dopamine (DA) indirect regulation of motor function through the modulation of basal ganglia activity has been widely described (Alexander et al., 1986; Lang and Lozano, 1998; Murer et al., 2002; Dejean et al., 2012). In addition, neuroanatomical studies have shown the existence of a direct DA innervation from the midbrain to M1 that could directly modulate M1 neuronal activity (Descarries et al., 1987; Gaspar et al., 1991; Raghanti et al., 2008).

Indeed, Gaspar et al. (1991) suggested the presence of such an innervation in the most superficial layers in human M1 using a tyrosine hydroxylase (TH) immunostaining to visualize monoaminergic fibers. In rats, Descarries et al. (1987) showed a dopaminergic innervation in cortical areas such as the cingulate cortex (Cg), or in the deep layers of M1, by using ^3^H-DA labeling. More recently, Hosp et al. (2011) described in rats direct projections from the ventral tegmental area (VTA) to M1. Although detectable dopaminergic tissue levels can be measured in the motor cortex, this DA innervation remains weak compared with other structures such as the striatum or nucleus accumbens. For instance, Godefroy et al. (1991) showed that DA concentration in the somatomotor cortex is about 50 times lower than in the striatum. However, the functional implication of DA in the motor cortex and other cortical regions, such as the prefrontal and cingulate cortices, has been well documented despite low tissue and extracellular DA levels (Awenowicz and Porter, 2002; Lopez-Avila et al., 2004; Schweimer and Hauber, 2006; Hosp et al., 2009; Molina-Luna et al., 2009). DA acts via five different receptors grouped in two classes, D_1_-like and D_2_-like, modulating differentially adenylyl cyclase (Jaber et al., 1996). In the last three decades, studies using *in situ *hybridization (Camps et al., 1990; Mansour et al., 1990; Gaspar et al., 1995; Santana et al., 2009) showed a wide distribution of the DA receptors in rodents. In the cortex, D_1_ receptors are localized in the layer VI whereas D_2_ receptors are localized primarily in the layer V (Weiner et al., 1991; Gaspar et al., 1995), which contains the principal output pathway to all other cortical areas and to subcortical targets as the striatum or the pyramidal tract. Taken together, these data suggest that DA receptors could play a direct role in modulating the activity of M1.

Awenowicz and Porter (2002) and Huda et al. (2001) described *in vivo*, respectively, in rats and cats, that DA application decreases pyramidal neurons activity via both D_1_ and D_2_ receptors. More recently, Hosp et al. (2009) showed a transient reduced excitability of M1 mediated by the injection of a D_2_ antagonist, but not a D_1_ antagonist, in rats *in vivo*. Moreover, specific dopaminergic deafferentation of M1 impairs motor skill learning (Hosp et al., 2011) and is associated with decreased long term potentiation (LTP) that is mimicked by reversible blockade of D_2_ receptors (Molina-Luna et al., 2009). These data suggest that D_2_ receptors could potentiate basal activity of M1 neurons. Even though a DA projection was reported in M1, the literature lacks quantification of this innervation. Moreover, functional studies are still conflicting regarding the involvement of D_1_ receptors in the modulation of M1 neuronal activity, and even though the literature agrees on the involvement of D_2_ receptors, results diverge regarding its excitatory or inhibitory effect on M1 activity. Unfortunately, none of these studies was performed in mice; this is of interest given the substantial number of transgenic mice models targeting the DA system and often used as models of psychiatric or neurodegenerative disorders.

The aim of this study was to assess the neuroanatomical distribution of DA innervation in M1 in mice, and to evaluate the functional role of this innervation on M1 neuronal activity. To this end, we first characterized anatomically DA fiber density in M1 by using the DA transporter (DAT) as a specific marker of DA terminals. In order to precisely quantify this innervation, we performed an unbiased stereological quantification of DAT labeled fibers in M1. Secondly, since all previous studies consensually point to an involvement of D_2_ receptors in M1, we have tested the direct influence of DA on M1 neuronal activity through this receptor. For that purpose, we performed electrophysiological recordings of M1 neuronal activity while pharmacologically modulating D_2_ receptors. Our study indicates that DA innervates M1 in mice and is able to enhance the activity of pyramidal neurons in this structure.

## MATERIALS AND METHODS

### ANIMALS AND SURGERY

All experiments were conducted in accordance with the guidelines of the French Agriculture and Forestry Ministry (decree 87849) and of European Union Directive (2010/63/EU). Adequate measures were taken to minimize animal pain as well as the number of animals used. Female mice C57/BL6 (3–6 months at the time of experiments, Janvier, France) were housed in ventilated cages and kept under a 12 h dark/light cycle. Animals had access to food and water *ad libitum*.

Before surgery, mice were deeply anesthetized with Urethane (1.8 g/kg) injected intraperitoneally (i.p.) before being secured to a stereotaxic frame (LPC, France) and maintained at 37–38°C with a heating pad. A mouse brain stereotaxic atlas (Paxinos and Franklin, 2001) was used to guide electrode and pipette placements. Throughout the experiment, the efficiency of anesthesia was determined by examining the tail pinch reflex. Additional Urethane (0.25 g/kg, i.p.) was administered when necessary.

### ELECTROPHYSIOLOGICAL PROCEDURES

Electrophysiological single unit activity was recorded in M1 using electrodes pulled from borosilicate glass capillaries (GC 150 F, Harvard Apparatus, England) with a P-97 Flaming Brown (Sutter Instrument, USA). The tip of the electrode was broken to a diameter of 2 μm, and the electrode filled with a 0.4 M NaCl solution containing 2.5% neurobiotin (Vector Labs, USA). Electrodes had an *in vivo* resistance of 12–20 MΩ. Recording electrodes were lowered in M1 (1.3–1.5 mm lateral and 1.0–1.5 mm anterior to bregma) at a depth of between 0.65 and 1 mm from the brain surface.

Neuronal activity was amplified 10 times, filtered (bandwith: 300 Hz–10 kHz), and further amplified 100 times (Multiclamp 700-B, Axon Instruments, USA). The signal was digitized (Micro 1401 mk II, Cambridge Electronics Design, England) and acquired on computer using Spike 2 software. Recorded neurons were juxtacellularly labeled with neurobiotin (Vector Labs, USA) as described elsewhere (Pinault, 1996). Briefly, positive 250 ms current pulses were applied at 2 Hz with increasing currents (1–5 nA) until driving cell firing for at least 5 min. Immediately after the neurobiotin injection, mice were transcardiacally perfused with 0.9% NaCl and 4% paraformaldehyde (PFA). Brains were collected and post-fixed for 24 h at 4°C in 4% PFA and cryoprotected overnight in 30% saccharose at 4°C. Serial coronal sections (40 μm) containing M1 were cut using a cryostat (CM 3050 S, Leica, Germany). To reveal neurobiotin, sections were rinsed three times in 0.1 M phosphate buffer saline (PBS), processed for 1 h with a blocking solution (3% bovine serum albumine (BSA), 0.3% Triton X-100 in PBS) and incubated overnight at 4°C within Streptavidin Alexa 568 (Invitrogen, USA) diluted 1:800 in PBS containing 3% BSA and 0.3% Triton X-100. Sections were then rinsed three times in PBS before being mounted on gelatin coated-slides, air-dried and coverslipped with DePeX (VWR, USA).

Antidromic stimulation of the striatum ipsilateral to the recording site was performed using a concentric bipolar electrode (SNEX-100, Rhodes Medical Instruments, USA) implanted in the dorsolateral striatum (2 mm lateral and 0.2 mm anterior to the bregma, depth of 1.85 mm from the brain surface). Electrical stimulations (0.5 ms, 600–800 μA) were applied every 5 s using an external stimulator (DS3; Digitimer, England) triggered by a 1401 Plus system (Cambridge Electronic Design, England).

### DRUG APPLICATION

Systemic administration of D_2_ pharmacology was performed through an i.p.-implanted-needle connected to a syringe filled either with a D_2_ agonist (quinpirole, 0.5 mg/kg, Sigma, USA), D_2_ antagonist (haloperidol, 0.5 mg/kg, Sigma, USA) or 0.9% NaCl. Drug injections were performed after a 30 min baseline recording and electrophysiological activity was monitored for 45 min following the injection.

Local intracortical drug administration was performed using a glass pipette pulled from a glass capillary (GC 100 FS, Harvard Apparatus, England) filled with either quinpirole 100 μM, quinpirole 1 μM or artificial cerebrospinal fluid (ACSF) that was lowered close to the tip of the recording pipette. After a 5 min baseline recording, the drug was applied by air pressure and neuronal firing was monitored for another 15 min.

### ANALYSIS OF ELECTROPHYSIOLOGICAL DATA

The recordings were analyzed offline. Action potential (AP) duration was measured from the time when AP begins to the time when baseline is recovered. In order to assess the pharmacological modulation of neuronal activity, AP firing rate was analyzed before and after pharmacological treatments of 10 min or 1 min durations, respectively, for i.p. and intracortical drug injection. AP durations, neuron responsiveness to striatal stimulation, and firing frequencies were analyzed using Spike 2 7.0 (Cambridge Electronics Design, England). AP firing patterns were analyzed using NeuroExplorer burst analysis (maximum interval to start a burst = 40 ms, maximum interval to end a burst = 10 ms, minimum interval between bursts = 20 ms, minimum duration of a burst = 5 ms and minimum number of spikes in a burst = 2).

### IMMUNOHISTOCHEMICAL PROCEDURES

Three mice were deeply anesthetized with chloral hydrate (400 mg/kg). They were then perfused transcardiacally with 0.9% NaCl and 1% PFA. Brains were removed, post-fixed in 1% PFA at 4°C for 24 h and cryoprotected overnight in 30% saccharose. Brains were serially cut in six sets of coronal sections (40 μm) using a vibrating microtome (MICROM HM 650 V, Thermo Scientific, France). Free-floating sections were kept at -20°C in glucose 0.19%, ethylene glycol 37.5% and sodium azide 0.25% in PBS 0.05 M.

For each brain, one of the six sets of sections was randomly chosen for DAT immunohistochemical processing. Sections were rinsed three times in 0.1 M Tris-buffered saline (TBS), treated with 0.6% H_2_O_2_ in TBS for 15 min, rinsed three times in TBS, and incubated for 90 min in blocking solution (10% donkey serum, 0.3% triton X-100 in TBS). Sections were incubated for 48 h at 4°C with primary antibody (rabbit anti-DAT, 1:5000, gift from Pr Bertrand Bloch, CNRS UMR5293) diluted in blocking solution. Sections were rinsed three times in TBS and incubated for 1 h in the secondary antibody (donkey anti-rabbit biotin SP, Jackson Immuno Research, USA) diluted 1:500 in TBS containing 5% donkey serum and 0.3% triton X-100. Sections were rinsed three times in TBS, incubated in 0.5% avidin–biotin complex (Vector Labs, USA) in TBS, rinsed three times in TBS and processed with 3-3′-diaminobenzidine (Sigma, USA) and 0.33% H_2_O_2_. Sections were mounted, air-dried, and coverslipped in DePeX (VWR, USA).

### STEREOLOGICAL ANALYSIS

Cingulate cortex was defined anteriorly from 2.58 mm to the bregma to posteriorly -0.82 mm to the bregma, as defined by Paxinos and Franklin (2001). The medial boundaries are defined by the medial line of the brain and the lateral boundaries are defined by the presence of horizontal cortical layers. M1 was defined anteriorly from 1.1 mm bregma to posteriorly -0.94 mm to the bregma from layers I to VI, as defined in a stereotaxic atlas. The relatively narrow layer IV and thick layer V defined the lateral and medial boundaries of M1, and ventral boundaries consisted of the most dorsal part of the corpus callosum. The deep layers of M1 were defined as the most ventral half of M1 (from 500 μm to the surface to the dorsal outline of the corpus callosum), as defined by Lev and White (1997). For the total number of sections containing M1, we sampled every sixth section, starting with a section randomly selected from the first six sections, to generate a set of distributed sections within each sample. After the DAT immunohistochemistry, the average final thickness of the sections was 11.97 ± 0.38 μm (i.e., a shrinkage of ~70% during processing). The stereological analysis used was described previously by Mouton et al. (2002). Each section was scanned by a camera (Orca-R^2^, Hamamatsu Electronic, Japan) connected to a microscope (DM 5500, Leica, Germany). Then, virtual sphere probes were scanned on the *Z* axis of M1 and Cg using the Mercator Software (Explora Nova, France). Each sphere was 4 μm radius and contained in a 10 μm × 10 μm square, spacing between each square was 50 μm × 50 μm. Spheres were visualized as a series of concentric circles of changing circumferences upon focusing through the tissue. Finally, the intersections between the outline boundary of the sphere and the fibers were counted at each focal plane. To avoid artifacts due to border effects, upper and lower guard zones of 1 μm were kept for each section. The total length of fibers is calculated according to the following equation:

$$L=2\cdot \sum Q[v/a]F_{1}\cdot F_{2}\cdot F_{3}$$

where *L* = total length of linear feature (in μm), ∑*Q* = sum intersections between fibers and spheres, *F*_1_ = 1/section sampling fraction (1/6), *F*_2_ = 1/area sampling fraction, *F*_3_ = 1/thickness sampling fraction, *v*/*a* = the ratio of the volume of one sampling box to the surface area of one spherical probe. All values are given as the mean ± SE. Calculated values are corrected for the 70% shrinkage due to section processing.

### DETERMINATION OF THE DOPAMINERGIC FIBERS DISTRIBUTION WITHIN M1

To determine the rostrocaudal and mediolateral extent of dopaminergic fibers within M1, photomicrographs of sections that previously underwent stereological analysis were used to determine the surface area occupied by DAT labeled fibers. On each section, the results were plotted as the occupied surface in μm^2^ relative to the anteroposterior axis. Measures were performed using ImageJ 1.47v.

### STATISTICAL ANALYSIS

Statistical analyses were performed using the Mann–Whitney test for independent data, and a two-way ANOVA with Bonferroni posttests when comparing drugs effect over time.

## RESULTS

### ANATOMICAL DISTRIBUTION OF THE DOPAMINERGIC TERMINALS IN M1

**FIGURE 1:**
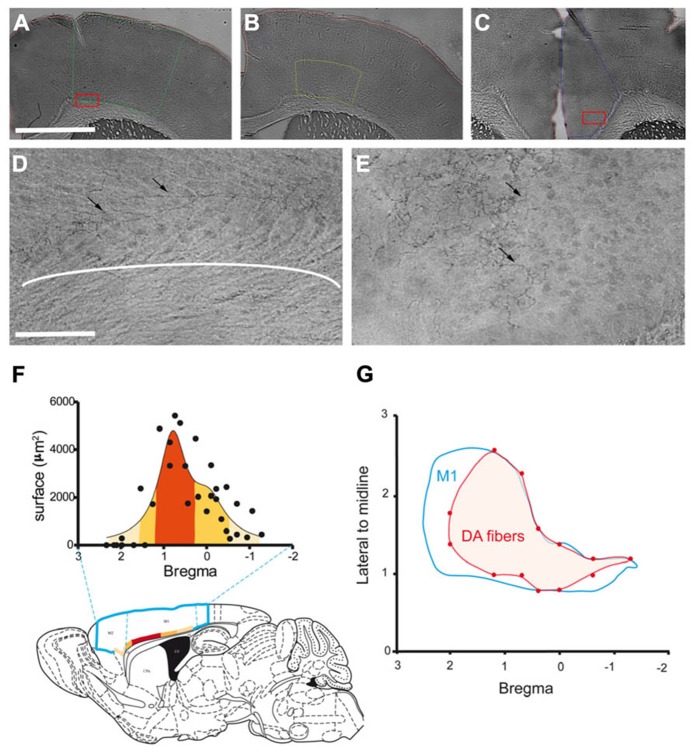
**Anatomical distribution of the dopaminergic terminals in M1.** **(A–C)** Example of the delimitation of cortical regions: M1 (**A**; green line), M1 deepest layers (**B**; yellow line) and Cg (**C**; blue line). **(D–E)** Photomicrographs of DAT immunostained fibers (black arrows) in M1 deepest layers **(D)** and Cg **(E)**. **(D)** and **(E)** were obtained from higher magnification of the region contained in the red box shown in **(A)** and **(C)**, respectively. **(F)** Rostrocaudal repartition of DAT immunostained fibers in M1, the labeled superficies are represented for each level according to Bregma. The colors from light to dark orange code for the size of the labeled area, this color code is used to represent on the schematic sagittal section of mouse brain the distribution of these areas in the motor cortices (blue line delineates M2 and M1). **(G)** Distribution of the DAT labeled fibers at the surface of the cortex. The blue line represents delimitation of M1, the red area represents DAT immunostained fibers within M1. Scale bars represent 100 μm **(A–C)** and 12.5 μm **(D–E)**.

DA fibers were labeled using DAT immunostaining in order to visualize the dopaminergic innervation in M1 (**Figures 1A,B,D**) and Cg (**Figures 1C,E**). Dopaminergic fibers were present in the deep layers of M1. In M1 and Cg, these fibers were long, tortuous and thin with tangles and branches. Stereology was used to precisely evaluate the extent of this innervation.

The mean total length of dopaminergic fibers was 1.89 ± 0.22 m in M1 and 3.64 ± 0.56 m in Cg. The dopaminergic innervation density, calculated as the result of the total fibers length divided by the volume of the structure, was 0.54 ± 0.01 m/mm^3^ in M1 and 2.18 ± 0.20 m/mm^3^ in Cg. Thus, according to this stereological approach, DA innervation is 4.4 times higher in Cg than in M1. However, since the dopaminergic fibers in M1 were found mostly in the deep layers (**Figure 1D**), we performed a stereological quantification of the dopaminergic innervation in the deep layers of M1 defined as the deepest half of M1 (**Figure 1B**). Total dopaminergic fibers length in the deep layers of M1 was 1.39 ± 0.06 m. This length is not statistically different from the total length of dopaminergic fibers found in the entire volume of M1 (*p* = 0.097), confirming our initial observation that dopaminergic terminals in this structure are mostly restricted to the deep cortical layers. The density of DA terminals in the deep layers of M1 was then estimated to 1.38 ± 0.17 m/mm^3^. Therefore, when restricting the analysis to the specific region innervated by DA in M1, the dopaminergic innervation density is of the same order of magnitude as in Cg.

To further characterize the neuroanatomical distribution of dopaminergic innervation, we measured the distribution of DA fibers within M1. Differences appeared in the rostrocaudal distribution of DA fibers. Indeed, the area innervated by DA fibers is maximal between 0.2 and 1.10 mm anterior to the bregma (**Figure 1F**). Furthermore, regarding the mediolateral distribution of dopaminergic fibers in M1 (**Figure 1G**), we observed that only this area, which corresponds to the forelimb representation area (Tennant et al., 2011), is innervated on the whole mediolateral extend of the structure.

Altogether, these data show that DA innervates the deep layers of mouse M1 with a rostrocaudal gradient. The density of this innervation in M1deep layers is comparable to that of Cg. It has been well described that DA could modulate Cg neuronal activity (Lopez-Avila et al., 2004; Schweimer and Hauber, 2006). Thus, our results further suggest that the density of DA innervation in M1 deep layers could be sufficient to significantly impact neuronal activity.

### ELECTROPHYSIOLOGICAL CHARACTERISTICS OF RECORDED NEURONS

**FIGURE 2:**
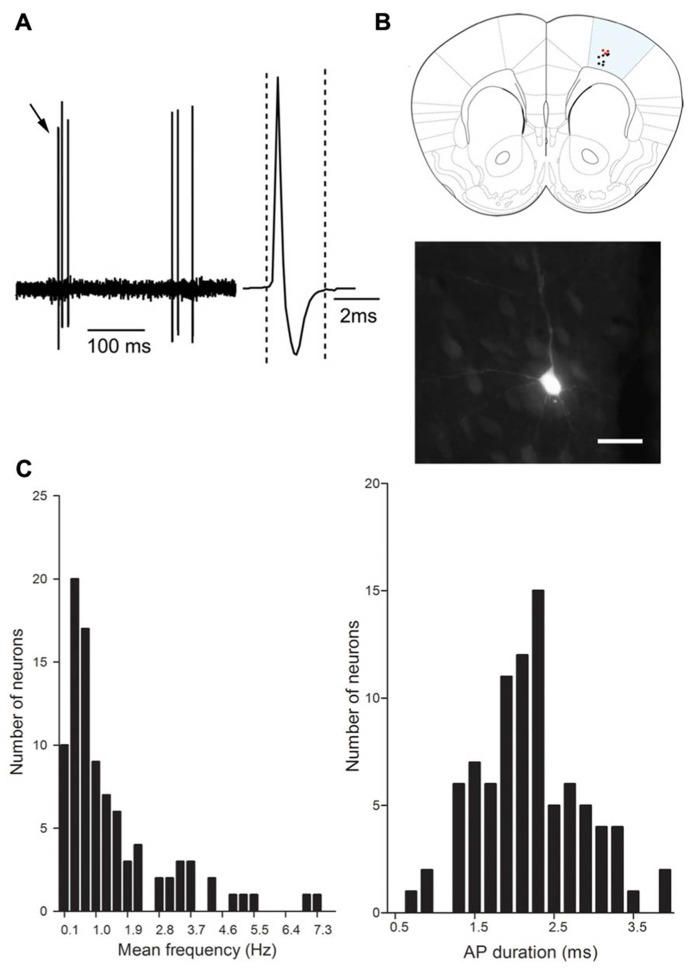
**Electrophysiological characteristics of M1 neurons. (A)** Representative electrophysiological trace of a cortical neuron. Note the presence of triplet of spikes (black arrows). The inset represents the action potential shape (averaged over 5 min recording), the action potential duration is measured between the two dashed lines. **(B)** Schematic representation of the distribution of recorded neurons in M1 1.4 mm anterior to Bregma, neurobiotine labeled neurons (red dots) and non labeled neurons (black dots). Photomicrograph shows a representative example of neurobiotine labeled neuron. Scale bar represents 20 μm. **(C)** Distribution of the mean frequency (Hz) and AP duration (ms).

**FIGURE 3:**
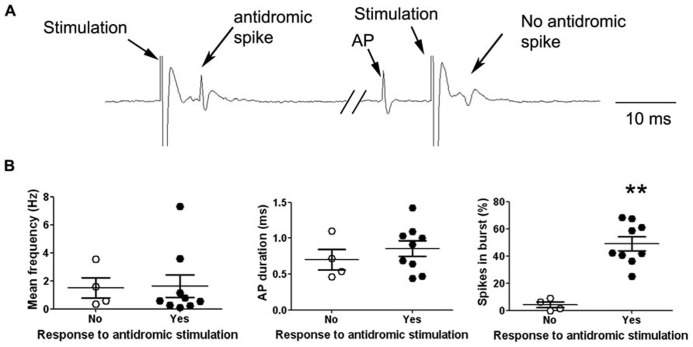
**Electrophysiological characteristics of antidromically identified neurons.** **(A)** Representative electrophysiological recording trace of a cortical neuron responding to the striatal stimulation by an antidromic spike (left). The occurrence of a spontaneous AP just before the stimulation collides with the antidromic spike resulting in the absence of the antidromic response after the stimulation (right). **(B)** Neurons were divided in two groups according to their response (black dots) or not (white dots) to the striatal stimulation, the graphs show the individual data (large dots) as well as the mean ± SEM of electrophysiological characteristics: Mean frequency (Hz), AP duration (ms) and percentage of spikes included in a burst (%). **p < 0.01.

We addressed the functional role of D_2_ receptors on M1 neuronal activity by electrophysiological single unit recordings in anesthetized mice (**Figure 2A**). Ninety-seven neurons in 56 mice were recorded in deep layers (**Figure 2B**). In order to investigate D_2_ effects on M1 output neurons, we focused our experiments on pyramidal neurons, although local-circuit inhibitory neurons are also present (Markram et al., 2004). Previous studies have established the electrophysiological characteristics of pyramidal neurons in rat prefrontal cortex (PFC). Pyramidal neurons exhibit low firing frequencies (between 0.1 and 5 Hz; Hajos et al., 2003) and AP durations above 0.95 ms (Mallet et al., 2005; Tseng et al., 2006). We analyzed these physiological characteristics in the 97 neurons recorded in this study; however, in our conditions, no clear bi-modal distribution emerged from this analysis that would have allowed to discriminate between cortical neuronal populations (inhibitory interneurons and excitatory pyramidal neurons; **Figure 2C**). Regarding firing patterns, we found that 83 neurons presented doublets or triplets (**Figure 2A**) and a bursty discharge pattern (34.47 ± 2.44% of spikes in burst). In order to determine an inclusion criteria specific to our experimental conditions, we analyzed the electrophysiological characteristics of neurons identified as projection neurons by their antidromic response to the stimulation of the ipsilateral striatum (**Figure 3A**). Neurons that presented antidromic responses were considered as pyramidal. We recorded nine antidromically responding neurons and four neurons that did not respond to the striatal stimulation. Responsive and non-responsive neurons were statistically different regarding their firing pattern (*p* < 0.01). Indeed, all neurons responding to the antidromic stimulation presented at least 25% of their spikes in bursts (ranging from 25 to 68%) whereas the non-responding neurons presented at most 8.8% of their spikes in bursts (ranging from 0 to 8.8%; **Figure 3B**). Thus, in our experimental conditions, the percentage of spikes in bursts is the best electrophysiological characteristic to consider a neuron as a pyramidal one. Using this characteristic as a criterion, 30 neurons presenting at least 15% spikes in burst were included in the study and referred to as “putative pyramidal neurons”.

### EFFECTS OF DOPAMINE D_2_ RECEPTOR AGONIST AND ANTAGONIST ON PUTATIVE PYRAMIDAL NEURON ACTIVITY IN M1 *IN* VIVO

**FIGURE 4:**
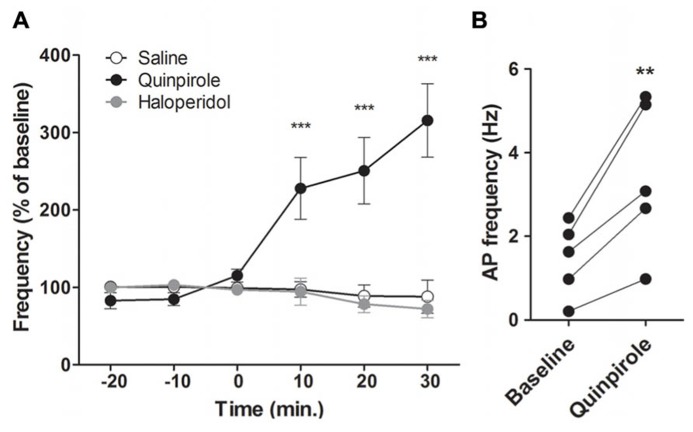
**D_2_ modulation of M1 neuronal activity. (A)** Effect of a peripheral injection of D_2_ agonist quinpirole (*n* = 5, black) or D2 antagonist haloperidol (*n* = 6, grey) or NaCl 0.9 % (*n* = 5, white) on putative pyramidal neurons firing frequency. **(B)** Individual responses of putative pyramidal neurons to the D2 agonist. **p < 0.01; ***p < 0.001.

To study the effects of DA on M1 neuronal activity, we recorded AP firing rate of putative pyramidal neurons in the deep layers of M1 and their response to the D_2_ agonist quinpirole or the D_2_ antagonist haloperidol. We first performed intraperitoneal (i.p.) injections of quinpirole (0.5 mg/kg; *n* = 5), haloperidol (0.5 mg/kg; *n* = 5) or saline 0.9% (*n* = 5; **Figure 4**). D_2_ receptor activation by quinpirole enhanced putative pyramidal neurons firing rate by more than 200% (from 1.46 ± 0.39 Hz to 3.44 ± 0.81 Hz, two way ANOVA *F*_(2,60)_ = 15.11, *p* < 0.001). There was no statistically significant effect of D_2_ receptors blockade by haloperidol on AP firing rate.

**FIGURE 5:**
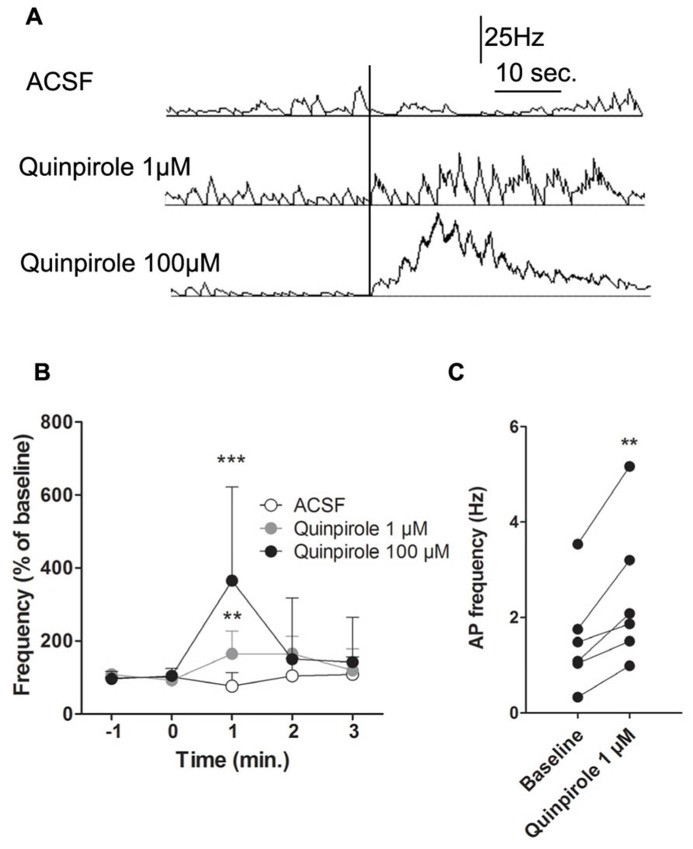
**Intracortical injection of D_**2**_ agonist quinpirole or ACSF. (A)** Typical recording of a putative pyramidal neuron 30 s before and 40 s after an injection of ACSF (upper panel), 1 μM (middle panel) or 100 μM quinpirole (lower panel). **(B)** Effect of an injection of ACSF on the mean AP firing frequency of putative pyramidal neurons (*n* = 5) in white. Effect of an injection of 100 μM quinpirole on the mean AP firing frequency of putative pyramidal neurons (*n* = 4) in black. Effect of an injection of 1 μM quinpirole on the mean AP firing frequency of putative pyramidal neurons (*n* = 6) in grey. **(C)** Individual responses of putative pyramidal neurons to 1 μM quinpirole. **(A)** The vertical black line represents the injection. **(B)** **p < 0.01; ***p < 0.001.

These effects could be due to a network effect, particularly via the basal ganglia. To avoid the indirect network effects of DA and address the direct effect of D_2_ activation on M1 activity, we performed intracortical injections of quinpirole 100 μM, quinpirole 1 μM or ACSF (**Figures 5A,B**). Due to absence of significant modifications after i.p. injections of haloperidol, we did not test the pyramidal neuron responses to intracortical injections of the D_2_ antagonist. Consistent with the results obtained after i.p. injections, local D_2_ receptor activation by quinpirole (100 or 1 μM) enhanced putative pyramidal neurons firing rate (respectively: Two way ANOVA *F*_(4,28)_ = 5.24, *p* < 0.001; Two way ANOVA *F*_(__4__,36)_ = 3.98, *p* < 0.01). Quinpirole (1 μM) also increased spike firing rates from 1.53 ± 0.44 Hz to 2.47 ± 0.62 Hz (**Figure 5C**). Furthermore, analysis of neuronal AP firing pattern revealed that the number of bursts, but not the percentage of spikes in burst, was increased by D_2_ receptors activation (data not shown). These results indicate that DA can enhance pyramidal neuron firing rates, but does not modulate firing patterns. Taken together, these results show that DA exerts a direct role on M1 neuronal activity by enhancing neuronal firing rate via D_2_ receptors.

## DISCUSSION

In this study, we demonstrated for the first time in mice that DA innervates the deep layers of M1. We also established that these fibers target preferentially the forelimb representation area of M1. To address the functional role of DA on M1 neuronal activity, we performed electrophysiological recordings of single neuron activity *in vivo* and pharmacologically modulated D_2_ receptors. We demonstrated that D_2_ receptor activation by quinpirole enhanced pyramidal neuron spike firing rates. Our results also show that this increase was not due to an extracortical network effect, but is locally mediated in M1.

### ANATOMICAL CHARACTERIZATION OF DA INNERVATION OF M1 IN MICE

Although TH immunolabeling is commonly used to reveal dopaminergic fibers (Gaspar et al., 1991; Busceti et al., 2008), TH is an enzyme common to all catecholamines synthesis, and such does not allow one to distinguish between adrenergic and dopaminergic fibers. Thus, to specifically target dopaminergic fibers, we used a DAT antibody. DAT distribution has already been shown to be restricted to dopaminergic regions (Ciliax et al., 1995). Our results in mice showing the existence of a dopaminergic innervation of M1 are in accordance with previous studies conducted in different species including rat (Descarries et al., 1987), monkey (Raghanti et al., 2008) and human (Gaspar et al., 1991; Raghanti et al., 2008). Moreover, this study provides for the first time a precise and direct quantification of this innervation in M1 and Cg using an unbiased stereological approach. This quantification allowed us to precisely detail the distribution of DA fibers at different levels of M1. Our data complement previous observations by showing that the density of dopaminergic innervation is similar in the deep layers of M1 and in Cg. The functional significance of DA in Cg has been well established (Lopez-Avila et al., 2004). Previous studies showing the existence of D_1_ and D_2_ receptors in M1 (Camps et al., 1990; Mansour et al., 1990; Gaspar et al., 1995; Santana et al., 2009), together with our present results, provide anatomical evidence suggesting that DA can exert a direct influence onto M1 neuronal activity.

### DA MODULATION OF M1 NEURONAL ACTIVITY *IN VIVO*

We investigated the hypothesis that DA directly modulates M1 activity using single unit electrophysiological recordings in anesthetized mice and showed that DA has a direct influence on putative pyramidal neuron activity in M1. In our experiments, D_2_ receptor activation increased neuronal spike firing rate by enhancing the number of spikes, but not the percentage of spikes in bursts. Our results are consistent with a previous study showing in rats that a local injection of haloperidol induced an increase of motor threshold and a reduced size of motor maps, suggesting an excitatory role of D_2_ receptor activation in M1 (Hosp et al., 2009).

Awenowicz and Porter (2002) previously reported the involvement of the two types of DA receptors in a synergistic manner in rat motor cortex. Their study showed a global inhibitory effect in pyramidal neuron activity following iontophoretic DA (0.1 M) administration. The discordance between their results and ours could be explained by the difference in the local injection procedure (iontophoresis versus pressure ejection). Although this study showed a DA effect on M1 electrophysiological activity, one must consider the possible electrophysiological perturbations in neuronal activity induced by iontophoresis injection. Indeed, it was recently shown that high current injections near neurons can lead to decreased neuronal firing rates (Moore et al., 2011).

Our results showing enhanced putative pyramidal neuron activity after D_2_ receptor activation are consistent with the finding that quinpirole acting on D_2_ receptors increases the excitability of layer V pyramidal neurons in the PFC of adult mice (Gee et al., 2012). This study, performed in brain slices, demonstrated an excitatory effect of D_2_ receptor activation on PFC pyramidal neurons by the induction of a calcium-channel-dependent after-depolarization.

However, other scenarios might also contribute to the effects of D_2_ agonists on motor cortex excitability. On one hand, DA effects on putative pyramidal neuron activity might be local, but indirect via the modulation of cortical inhibitory interneurons. Indeed, in primate PFC, DA axons establish direct contacts with interneurons expressing parvalbumin (Sesack et al., 1998). More recently, Santana et al. (2009) reported that inhibitory interneurons in rats PFC express D_1_ and D_2_ receptors. Moreover, electrophysiological studies from mice and rat PFC slices suggest that D_2_ receptor activation inhibits GABA interneurons (Xu and Yao, 2010), resulting in a decreased GABA release probability and a reduction of inhibitory postsynaptic currents (Seamans et al., 2001). Although these studies were conducted in prepubertal animals, they suggest that D_2_ receptor agonists could decrease the activity of inhibitory interneurons, thus indirectly enhancing pyramidal neuron activity.

On the other hand, DA effects observed in this study might be exerted directly on pyramidal neurons. Indeed, a recent study in PFC showed that pyramidal neurons in rats express the D_2_ receptor mRNA (Santana et al., 2009). Thus, DA may directly enhance pyramidal neuron activity by activating D_2_ receptors.

Additionally, our pharmacological data cannot rule out an effect of D_2_ agonists on D_2_ autoreceptors on dopaminergic terminals. The presynaptic modulation of DA release by D_2_ agonists might induce postsynaptic D_1_ as well as D_2_ receptor modulation. However, in our conditions, since the D_2_ agonist would directly stimulate the postsynaptic D_2_ receptors, the presynaptic inhibition of DA release would mainly result in a decrease of D_1_ receptors stimulation.

### FUNCTIONAL AND PATHOLOGICAL CONSIDERATIONS

Finally, it is interesting to note that our study shows that DA innervation in mouse M1 specifically targets an area that corresponds to the forelimb representation (Tennant et al., 2011). DA in motor cortex is known to regulate novel motor skill learning (Molina-Luna et al., 2009; Hosp et al., 2011). Furthermore, recent studies in rats showed that unilateral disruption of DA projections to M1 leads to a reduction of forelimb representation map associated with a reduction of intracortical microstimulation-induced distal forelimb movements (Viaro et al., 2011) and impairs motor skill learning (Molina-Luna et al., 2009; Hosp et al., 2011). Thus, these studies suggest a potential role of DA in the modulation of forelimb representation in M1. Considering pathological conditions, patients with* de novo *Parkinson’s disease (PD), a neurodegenerative disorder caused mainly by disruption of the DA nigrostriatal pathway, show abnormally high grip force in a precision lifting task (Fellows and Noth, 2004). Moreover, Gaspar et al. (1991) have shown that PD patients have altered dopaminergic innervation of motor cortex. Disruption of fine motor skills may involve the degeneration of dopaminergic terminals in M1. Taken together, these results suggest a role for DA in fine motor skill control of forelimb. Interestingly, studies on human M1 also reported that LTP cannot be induced in PD patients (Morgante et al., 2006) as long as they are off dopaminergic medication (Huang et al., 2011). Furthermore, Morgante et al. (2006) indicated that abnormal motor cortex plasticity may underlie the development of L-DOPA induced dyskinesia in PD patients. These results suggest that DA could be a key component in M1 plasticity.

## CONCLUSION

In conclusion, our study provides for the first time a precise description of the dopaminergic projections to M1 in mice, with a stereological quantification of DA innervation density and fiber distribution within M1. In addition, we show an increased putative pyramidal neurons firing activity induced by local D_2_ agonist. The exact mechanisms of this modulation remain to be elucidated and the role of D_1_ receptors has yet to be considered. Nevertheless, these results constitute a new step towards understanding the mechanisms by which DA modulates M1 activity and suggest that altered local D_2_ modulation may be involved in pathophysiological conditions associated with disturbed DA homeostasis.

## Conflict of Interest Statement

The authors declare that the research was conducted in the absence of any commercial or financial relationships that could be construed as a potential conflict of interest.
